# The longitudinal urban cohort ageing study (LUCAS): study protocol and participation in the first decade

**DOI:** 10.1186/1471-2318-12-35

**Published:** 2012-07-09

**Authors:** Ulrike Dapp, Jennifer Anders, Wolfgang von Renteln-Kruse, Stefan Golgert, Hans Peter Meier-Baumgartner, Christoph E Minder

**Affiliations:** 1 Albertinen-Haus Geriatrics Centre, Scientific Department at the University of Hamburg, Sellhopsweg 18-22, D-22459, Hamburg, Germany; 2 Horten Zentrum, Medical Faculty, University of Zurich, Pestalozzistrasse 24, CH-8091, Zürich, Switzerland

**Keywords:** Longitudinal, Cohort, Health, Promotion, Frailty, Comprehensive assessment, Ageing, Elderly, Functional decline

## Abstract

**Background:**

We present concept, study protocol and selected baseline data of the Longitudinal Urban Cohort Ageing Study (LUCAS) in Germany. LUCAS is a long-running cohort study of community-dwelling seniors complemented by specific studies of geriatric patients or diseases. Aims were to (1) Describe individual ageing trajectories in a metropolitan setting, documenting changes in functional status, the onset of frailty, disability and need of care; (2) Find determinants of healthy ageing; (3) Assess long-term effects of specific health promotion interventions; (4) Produce results for health care planning for fit, pre-frail, frail and disabled elderly persons; (5) Set up a framework for embedded studies to investigate various hypotheses in specific subgroups of elderly.

**Methods/Design:**

In 2000, twenty-one general practitioners (GPs) were recruited in the Hamburg metropolitan area; they generated lists of all their patients 60 years and older. Persons not terminally ill, without daily need of assistance or professional care were eligible. Of these, n = 3,326 (48 %) agreed to participate and completed a small (baseline) and an extensive health questionnaire (wave 1). In 2007/2008, a re-recruitment took place including 2,012 participants: 743 men, 1,269 women (647 deaths, 197 losses, 470 declined further participation). In 2009/2010 n = 1,627 returned the questionnaire (90 deaths, 47 losses, 248 declined further participation) resulting in a good participation rate over ten years with limited and quantified dropouts. Presently, follow-up data from 2007/2008 (wave 2) and 2009/2010 (wave 3) are available. Data wave 4 is due in 2011/2012, and the project will be continued until 2013. Information on survival and need of nursing care was collected continuously and cross-checked against official records. We used Fisher’s exact test and t-tests. The study served repeatedly to evaluate health promotion interventions and concepts.

**Discussion:**

LUCAS shows that a cohort study of older persons is feasible and can maintain a good participation rate over ten years, even when extensive self-reported health data are collected repeatedly through self-filled questionnaires. Evidently individual health developments of elderly persons can be tracked quantifying simultaneously behaviour, co-morbidity, functional competence and their changes. In future, we expect to generate results of significance about the five study aims listed above.

## Introduction

Industrialized and developing countries around the world are ageing rapidly and Germany is among the countries at the top of this trend [1]. These countries will experience a rapid increase in both the number and proportion of old and oldest people in the population. However, little is known about the ageing process, life events and approaches that address health protection in old age [2]. Recent studies indicate that with the increase in the population proportion of old and very old people, the number of disabled persons will increase, as will the toll from chronic diseases. This probably will occur not withstanding improvements in disability prevention [3,4].

Within the field of functional disability research, two general areas stand out: studies of health promotion and disease prevention in older persons, and studies to intervene in complex geriatric syndromes. In many studies, the focus is on physical activity and functional competence in old age because physical activity is a key component under personal responsibility with regard to health and independence until the end of life. Functional competence is enhanced through increasing physical activity. Therefore physical activity and mobility are central components of the empowerment strategies used in programs of health promotion [5,6].

As well, little is still known about the early stages of functional changes as reflected by decreased performance, loss of competence (mobility-disability) and its impact on everyday life [7]. Continuous observation of elderly people over time has the potential to shed light on the disablement process [8,9]. Medical knowledge about ageing processes and functional decline originates mostly from studies in hospitals and nursing homes. However, those studies do not represent the large and growing population of community-dwelling persons who constitute the majority of elderly people [10]. New interdisciplinary approaches are needed to document the ageing process and health status of these persons, including the development of frailty and disability. Such information is needed to address the heterogeneous health and mobility needs of the community-dwelling elderly and to develop specific preventive and health care programs for these target groups [11-13]. The LUCAS project is designed to increase knowledge in the areas of frailty and disability and its’ prevention as a contribution to the framework of Bergman and colleagues [14].

### The LUCAS Consortium

Four hundred thousand persons aged 60 years and older live in the greater Hamburg area. Because of the complexity of ageing issues and their consequences for health care a multidimensional and interdisciplinary approach appears to be mandatory [15]. Therefore, a number of studies were started in 2007 under the umbrella of LUCAS in the area of Hamburg, Germany (http://www.geriatrie-forschung.de). The interdisciplinary LUCAS Consortium incorporated specialists from the city government, from Hamburg University and from institutions involved in serving the elderly in different settings [16]. All projects were grouped around a core project, the Longitudinal Urban Cohort Ageing Study, from which the acronym LUCAS was derived. This report presents the aims, methodology and selected characteristics of cohort participants of the LUCAS core study.

### The LUCAS core project: The longitudinal urban cohort

Our objective is to describe the research questions, structure and contents of the urban longitudinal cohort recruited in 2000/2001 and its 9-year follow-up. The concept was to establish a long-time cohort study of community-dwelling elderly to complement short-term studies dedicated to geriatric patients or studies on specific diseases. Our study design was influenced by longitudinal studies in the Netherlands (MAAS) [17], Italy (ILSA) [18,19], Spain (TSHA) [20], the United States of America (NMAPS) [21] and Canada (CSLA) [22]. An overview of longitudinal studies in elderly target populations is provided by the U.S. Institutes of Health, National Institute on Aging [23] and some are described in a review of longitudinal studies [24].

Community-dwelling older persons contributed questionnaire information at baseline (recruitment) in 2000/2001, shortly after recruitment (wave 1), 2007/2008 (wave 2) and 2009/2010 (wave 3). The questionnaires covered 18 health related domains, and included pre-clinical markers for functional decline, frailty and disability (Additional File 1: Table S1). At each wave, a set of core questions remained unchanged to investigate transitions between health stages. This information can be retrieved for every individual cohort member and every wave. With this information, cohort members can be classified according to their self-reported functional status at baseline, at waves 1, 2 and 3. Such data were used to determine subgroups for intervention studies in the other LUCAS subprojects, e.g. for clinical workups [25,26]. In addition, information on the time point and extent of care support needed as well as mortality was collected. The database contains records over time of 3,326 study participants and is continuously being updated; it will be continued until 2013 at least.

### Distinct characteristics of the LUCAS cohort

1. The LUCAS core study is a cohort study in which data were gathered from each individual cohort member at several time points. Therefore, it will permit to draw causal conclusions about health developments.

2. The LUCAS cohort started with independent community-dwelling elderly persons, excluding other groups such as the young, those suffering from terminal disease and disabled elderly persons.

3. The LUCAS cohort integrates medical, functional, psychosocial, biographical and nursing care aspects, collecting both quantitative and qualitative data suitable for analyses of change.

Major study aims are:

1. To describe and document certain aspects of the ageing process, e.g. the transitions from robust to frail or disabled health status. Factors influencing these transitions and sojourn times are of particular interest both for prevention and health-care services planning.

2. To find determinants of healthy ageing, based both on self-reported health information and preclinical and clinical markers obtained through medical examinations and comprehensive geriatric assessments (CGA) in subsets of the cohort selected according to self-reported functional status.

3. To measure the long-term effects of a health-promotion intervention that showed favourable results at 1-year follow up [27,28].

In the present report, we describe the participation of the study subjects and report selected health findings at baseline of the LUCAS longitudinal cohort.

## Methods/Design

Initial recruitment took place for the PRO-AGE study, the Hamburg arm of an EU-funded multi-centre project [27-30]. The Hamburg participants of the PRO-AGE study and their data served as the baseline of the LUCAS cohort. The LUCAS study was approved in 2000 and 2007 by the Ethics Committee of the General Medical Council (Ärztekammer) and by the Central Data Protection Agency, City of Hamburg. All records were made pseudonymous, all results anonymous. In 2000, participants were asked for permission to re-contact them in the future.

### Self-administered questionnaires

The data items collected during the first decade include socio-demographic data (age, gender, socio-economic status), diverse self-reported aspects of health such as self-perceived health, mood, memory, co-morbidity, pain, medication use, preventive care use (vaccinations and check-ups), functional status**,** falls and risk of falls*,* vision, hearing, oral health, physical activity, nutrition, alcohol use, tobacco use, means of transportation (walking, cycling, car driving, use of public transport), use of urban activity space and health behaviour attitudes (motivation and reasons for acceptance/rejection of health promoting and preventive interventions). These domains were selected in accordance with the results of a systematic review [31].

At baseline, the Pra-questionnaire augmented by a question about the need of human help in basic activities of daily life (ADL) was administered [32-34] followed by the HRA-O questionnaire [27,28] at wave 1. At waves 2 and 3, modified questionnaires were used still addressing all domains covered at wave 1. A set of core questions were kept unchanged and supplemented by questions suggested by recent research. An overview of the questions used in the various waves is given in the Additional File 1: Table S1.

### Recruitment process and data collection

**Figure 1:**
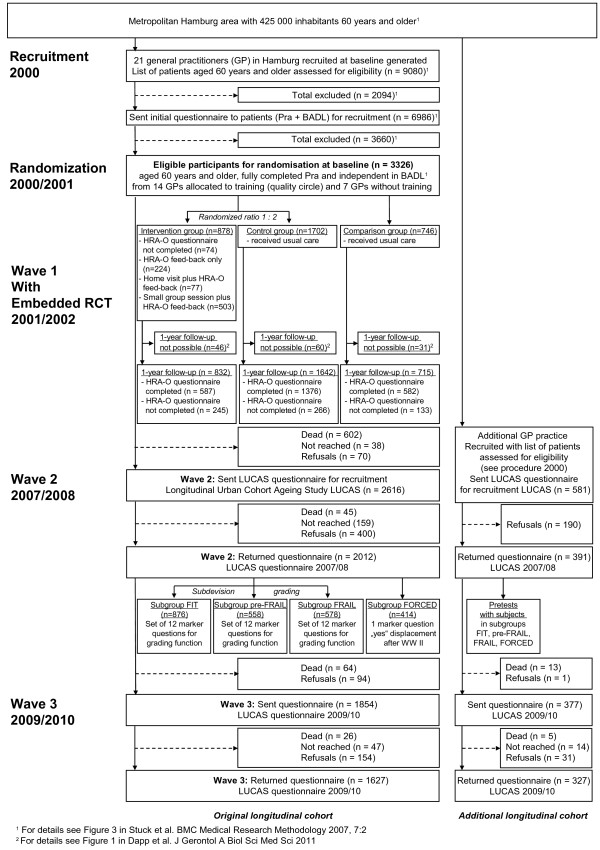
**Longitudinal urban cohort ageing study flow chart: The first ten years.**^1^ For details see Stuck and colleagues [27]. ^2^ For details see Dapp and colleagues [28].

Figure 1 depicts the flow of the recruitment of the study participants. In 2000, general practitioners (GPs) from the entire metropolitan area Hamburg were invited to participate in the newsletter of the regional GP association. Twenty-one GPs working in solo practices agreed to participate and were recruited. These GPs generated complete lists of all their patients aged 60 years and older. The GPs then excluded those patients with (a) a need of human assistance with ADL or needing professional nursing care (according to the German long-term care insurance system); (b) cognitive impairment (equivalent to a Mini Mental Status score ≤24 [35]; (c) terminal disease; and/or (d) inability to understand German.

#### Baseline (2000/2001)

All remaining 6,986 patients on the GPs´ lists were sent a letter of invitation, a consent form and the Pra-questionnaire with an additional question on the need of human help with ADL. Patients were asked to return the consent form and the completed baseline questionnaire if interested in participating in the study. Persons reporting a need of human assistance with ADL (84) and those returning an incomplete Pra-questionnaire or declining participation (3,576) were excluded from the study. The responses to the Pra-questionnaire of the remaining 3,326 study participants constitute the baseline of the LUCAS original cohort (Table 1, Figure 1).

**Table 1:** **Selected characteristics**^**1**^**of cohort participants at recruitment 2000/2001 and re-recruitment 2007/2008**

**Socio-demographic and health characteristics**^**1**^**at recruitment 2000/01**	**Participants at baseline n = 3326**	**Participants at wave 1 n = 2768**	**Participants at wave 2 n = 2012**	**All**^**2**^**non- participants at wave 2** **n = 1314**	**Of these: Dead**^**2**^**at wave 2** **n = 647**	**Of these: Refusers**^**2**^**at wave 2 ** **n = 470**	**Of these: ** **Lost to follow-up**^**2**^**at wave 2 n = 197**
**Age at baseline (years) –mean (min., max.)**	71.9 ± 7.8 (60.2-97.2)	71.2 ± 7.4 (60.2-97.0)	69.5 ± 6.5 (60.2-93.0)	75.5 ± 8.1 (60.3-97.2)	77.8 ± 8.0 (60.9-97.2)	72.5 ± 7.3 (60.9-95.8)	75.0 ± 8.3 (60.3-94.2)
**Female gender –** **n (in %)**	2126/3326 (63.9)	1728/2768 (62.4)	1269/2012 (63.1)	857/1314 (65.2)	361/647 (55.8)	347/470 (73.8)	149/197 (75.6)
**Fair/poor self-perceived health –n (in %)**	1290/3326 (38.8)	984/2768 (35.5)	623/2012 (31.0)	667/1314 (50.8)	364/647 (56.3)	213/470 (45.3)	90/197 (45.7)
**≥ 1 hospital admission over past 12 months – n (in %)**	714/3326 (21.5)	585/2768 (21.1)	394/2012 (19.6)	320/1314 (24.4)	193/647 (29.8)	87/470 (18.5)	40/197 (20.3)
**> 6 physician visits over past 12 months – n (in %)**	1615/3326 (48.6)	1342/2768 (48.5)	935/2012 (46.5)	680/1314 (51.8)	363/647 (56.1)	233/470 (49.6)	84/197 (42.6)
**Diabetes, yes –** **n (in %)**	356/3326 (10.7)	286/2768 (10.3)	183/2012 (9.1)	173/1314 (13.2)	106/647 (16.4)	45/470 (9.6)	22/197 (11.2)
**Coronary heart disease ever, yes – n (in %)**	571/3326 (17.2)	445/2768 (16.1)	288/2012 (14.3)	283/1314 (21.5)	167/647 (25.8)	83/470 (17.3)	33/197 (16.8)
**Heart attack ever, yes –n (in %)**	269/3326 (8.1)	204/2768 (7.4)	134/2012 (6.7)	135/1314 (10.3)	91/647 (14.1)	34/470 (7.2)	10/197 (5.1)
**Caregiver available if needed, yes –n (in %)**	2699/3326 (81.1)	2285/2768 (82.6)	1705/2012 (84.7)	994/1314 (75.6)	495/647 (76.5)	356/470 (75.7)	143/197 (72.6)

^1^Questions from Pra (Probability of Repeated Admissions) questionnaire [33,34].

^2^ All non-participants comprise those dead, refusing and lost to follow-up at wave 2.

#### Embedded randomised controlled trial (RCT)

The PRO-AGE Project which is at the base of the LUCAS cohort was an RCT designed to evaluate short-term effects of an intervention targeting preventive care and health behaviour described in [5,27,28,36-38]. Briefly, 2,580 study participants were randomised to intervention and control group while the remaining 746 participants constituted the comparison group. Intervention group members were given the HRA-O questionnaire immediately, received a computer-generated health report and were offered one of three health interventions: small group sessions, home visits or no health training. Control and comparison groups received the HRA-O questionnaire at year 1 (Figure 1).

From the perspective of the LUCAS cohort, the RCT described above is embedded into the cohort. This has several advantages: the continuing cohort allows an evaluation of the effectiveness of the health intervention over a longer time span. Second, the embedding into the cohort is crucial for the interpretation of the RCT results.

#### Wave 1

We defined “wave 1 results” as responses to HRA-O before any intervention. For the 804 responders of the intervention group, wave 1 data were collected just after randomisation in 2000/2001. Directly after finishing the one year intervention period, all 3,189 reachable surviving participants were sent a HRA-O questionnaire. Six members of the intervention group who had not responded HRA-O in 2000/2001 contributed a response in 2001/2002; they had not received an intervention. In addition, control (1,376 responders) and comparison groups (582 responders) received their HRA-O questionnaire in 2001/2002; thus their wave 1 data collection took place one year later. There were 804 + 6 + 1,376 + 582 = 2,768 wave 1 responses, 804 in 2000/2001 and 1,964 in 2001/2002 (Figure 1).

#### Wave 2

Wave 2 was started seven years after baseline to allow enough time for relevant and significant ageing effects to take place in the relatively young cohort (mean age at baseline 71.9 years). Some key questions of the HRA-O questionnaire were used again at wave 2 in 2007/2008. These key questions were supplemented by additional questions on psychological items, physical and mental activities, health literacy, income and use of urban activity space. Of the 3,326 persons at baseline, 647 had died and 197 were lost to follow-up. Therefore, 2,482/3,326 (74.6 %) participants of the original cohort could be contacted again and were sent a questionnaire. Completed questionnaires and written consent forms were returned by 2,012 original participants (81.1 %); 470 persons (18.9 %) refused further participation. To compensate for the 844 losses (one quarter since baseline), the cohort was supplemented by the eligible patients from an additional GP practice. Data from this group are not available at baseline (Figure 1, Table 1).

Table 1 shows the distributions of age, gender and health parameters according to participation status. At baseline, no participants were in need of help for daily activities or nursing care (exclusion criteria). Participants in wave 2 (2007/2008) were younger at baseline than those who died before wave 2 (t-test, p < 0.0001). The persons who refused and were lost to follow-up were older than the participants of wave 2 (t-tests, p < 0.0001). Men had a higher probability of dying than women (Fisher’s exact test [FET], p < 0.0005) and more women dropped out during the 7-year follow-up period (FET, p < 0.0005).

Those who died in the seven-year period before wave 2 had, at baseline, higher prevalence of fair/poor self-perceived health, of hospital visits and physicians visits in the year before baseline, of diabetes, self-reported coronary heart disease and heart attacks than wave 2 participants (FET, all p < 0.0005). At baseline, the prevalence of fair/poor self-perceived health was higher and the prevalence of potential caregivers lower among non-participants (dropouts or lost to follow-up) than participants of wave 2 (FET, both p < 0.0005).

**Figure 2:**
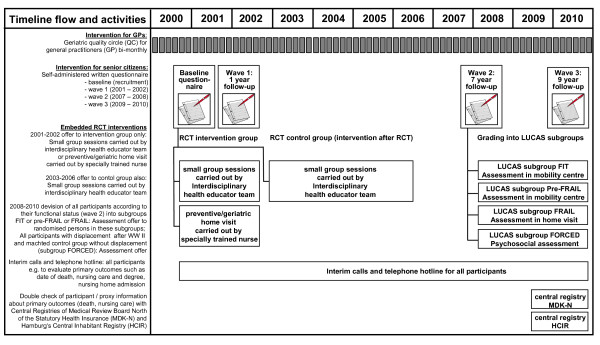
Longitudinal urban cohort ageing study time flow and intervention chart 2000–2010.

Between waves 2 and 3, embedded studies were set up to investigate various hypotheses. CGA data were collected by specialists from randomly selected LUCAS subgroup samples and analysed to strengthen the validity of instruments based on written self-reports (within-person analysis) (Figure 2).

#### Wave 3

The questionnaire was based on the wave 2 questionnaire and supplemented by questions on co-morbidity, cognitive activity, life course, social integration, assisted living, and mobility in urban activity space. The questionnaire was sent to the 1,854 remaining participants (64 dead, 94 refused), and 1,627 (87.7 %) of these returned a completed questionnaire (26 deaths, 47 lost to follow-up, 154 refused).

#### Continuous data collection

Starting in 2000, telephone interviews were used to collect information about reasons for refusal and the following outcomes (including their exact date): use of nursing care (ambulatory or institutional); eligibility of and intensity of nursing care assistance (“Pflegestufe”, according to the German long-term care insurance system) and death. Immediately before starting wave 3, the mortality data were verified and completed by information from the death registry. The nursing care and dependency data were also verified and completed by using data from the German long-term care insurance registry (MDK Nord, http://www.mdk-nord.de/)**.**

### Characteristics of participants and non-participants

There were no statistically significant differences in age and gender between participants and eligible persons who declined participation at baseline [28]. Furthermore, the LUCAS cohort population structure was comparable with the elderly population in the Hamburg city district where most of the GPs were located [39].

In Table 2, the results of the baseline Pra-questionnaire were compared according to participation in follow-ups**.** The Pra-questionnaire as well as waves 1 and 2 were answered by 1,892 of 3,326 participants; 876 responded to the Pra and wave 1 questionnaires only; 120 to the Pra and wave 2 questionnaires only; and 438 responded to the baseline Pra-questionnaire only but no further questionnaire (first line of Table 2). Non-response was caused by death, loss to follow-up and refusal. The 1,892 cohort members who had responded to all three questionnaires were younger, less frequently female (except Pra and wave 1 only responders), less frequently reported fair/poor self-perceived health, and less frequently suffered from diabetes, coronary heart disease and heart attack. They more often had a caregiver compared to the participants in the other groups. Excepted those persons who answered only at baseline and wave 2, the participants responding to both waves had experienced less frequently hospital admissions and six or more physician visits in the year before baseline.

**Table 2:** Baseline characteristics of participants according to further participation in waves 1 and 2

**Pra Characteristics at baseline (2000/2001)**	**Participation in**							
	**Wave 1**	**Wave 2**	**Wave 1**	**Wave 2**	**Wave 1**	**Wave 2**	**Wave 1**	**Wave 2**
	**Yes**	**Yes**	**Yes**	**No**	**No**	**Yes**	**No**	**No**
	**n = 1892**		**n = 876**		**n = 120**		**n = 438**	
**Age at base line (years) –**	69.4 ± 6.4		75.1 ± 7.8		71.5 ± 7.6		76.3 ± 8.7	
**mean (min., max.)**	(60.2-93.0)		(60.3-97.0)		(60.5-92.0)		(60.8-97.2)	
**Female gender –**	1185/1892		543/876		84/120		314/438	
**n (in %)**	(62.6)		(62.0)		(70.0)		(71.7)	
**Fair/poor self-perceived health –**	574/1892		410/876		49/120		257/438	
**n (in %)**	(30.3)		(46.8)		(40.8)		(58.7)	
**≥ 1 hospital admission over past 12 months –**	375/1892		210/876		19/120		110/438	
**n (in %)**	(19.8)		(24.0)		(15.8)		(25.1)	
**> 6 physician visits over past 12 months –**	883/1892		459/876		52/120		221/438	
**n (in %**	(46.7)		(52.4)		(43.3)		(50.5)	
**Diabetes, yes –**	167/1892		119/876		16/120		54/438	
**n (in %)**	(8.8)		(13.6)		(13.3)		(12.3)	
**Coronary heart disease ever, yes –**	268/1892		177/876		21/120		106/438	
**n (in %)**	(14.2)		(20.2)		(17.5)		(24.2)	
**Heart attack ever, yes –**	119/1892		85/876		15/120		50/438	
**n (in %)**	(6.3)		(9.7)		(12.5)		(11.4)	
**Caregiver available if needed, yes –**	1604/1882		671/876		91/120		323/438	
**n (in %)**	(85.2)		(76.6)		(75.8)		(73.7)	

### Statistical methods

Discrete outcomes were tabulated or percentages were given, tests were done using Fisher’s exact test (FET). Continuous outcomes were compared between groups using t-tests. Statistical analyses were done using SPSS, version 12 (SPSS GmbH Software, Munich) and Stata, version 10 (Stata Corporation, Texas).

## Discussion

**Figure 3:**
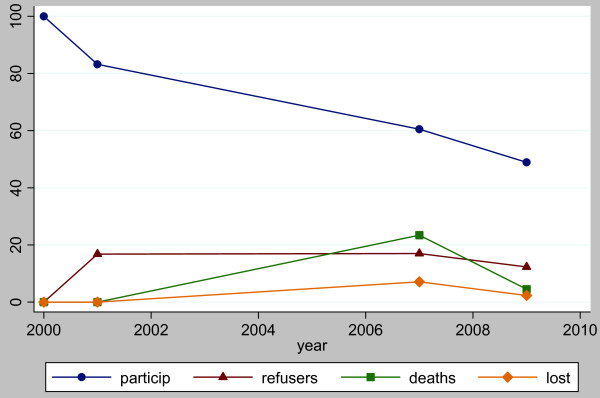
**Longitudinal urban cohort ageing study participation, refusals, deaths and losses over the first nine years. particip: percentage of participants remaining from baseline (n = 3,326).** refusers: percentage of persons who refused the current wave but had participated in the previous wave. deaths: percentage of persons who died between the previous and current wave. lost: percentage of persons who were lost to follow-up between the previous and current wave.

The LUCAS cohort focuses on the transition of self-supporting community dwelling elderly persons from a healthy, self-reliant initial state, through functional decline, frailty, disability, dependence and ultimately to death in a highly developed industrial country. To our knowledge there are only few comparable studies which monitor the ageing process of individuals without need of human assistance in basic activities of daily living at baseline (e.g. MNAPS [21]). Many longitudinal studies focusing on the health status in old age are population-based (e.g. ISLA [18]), random sampled (e.g. CLSA [22]) or participants are retrieved from disease-oriented registries (e.g. National cancer registries, German long-term care insurance registry). We demonstrated the feasibility of a cohort study in older persons. The key features were recruitment and training of general practitioners (GPs), access to participants through the GPs, and collecting self-reported data covering many aspects of health. The study did maintain a good participation rate over a ten-year period (Figure 3). Moreover, it appeared that distortions through dropout were limited and could be quantified (Table 2).

The scientific advantages of cohort studies are well known, e.g. [9,40]: causal inferences about determinants of health are made possible by observing the same individuals repeatedly over time. Our study was designed to assess multiple outcomes of primary and secondary hypotheses and was powered to address the most important questions. The multidisciplinary LUCAS team carefully selected outcomes with the aim to capture various combinations of aetiology, behaviour and culture.

We presented evidence that individual health development incorporating life-course perspectives and historical exposure information of ageing elderly can be tracked through self-filled questionnaires. In future, we expect to generate relevant results about ageing processes, and on predictors of healthy ageing.

Our chief aim is to better characterise normal ageing in order to generate new insights into the epidemiology of ageing. The LUCAS Consortium has a special interest in early signs of functional deterioration (frailty) [15], with the aim of reversing the process of deterioration. Thus, one area of work is to delineate screening tests for the onset of frailty suitable for routine use. The cohort study setting allows establishing predictive properties of screening instruments, similar to the proposal by Rockwood and colleagues [41] but based on self-reporting.

Table 1: Long-time participants were healthier and younger at baseline than non-participants. Participants who died within the first seven years of follow-up were the least healthy at baseline. Causes were again age and health differences, the less healthy being more prone to die. Some dropouts may have been due to patients having participated to please their doctor and then, not putting too much trust into prevention, stopped participation. Such considerations were reported by some dropouts. This interpretation is supported by the observation that in the 1,314 non-participants of wave 2 the prevalence of diabetes and coronary heart disease was about 50 % higher than in the 2,012 wave 2 participants. However, the prevalence of more than six doctor’s visits over the last year was only about 10 % higher, and that of hospital visits was even lower.

Table 2: The six self-reported health parameters show some of the health selection effects leading to non-participation. In general, the pattern found in this table appears to fit the hypothesis that persons less healthy at baseline were less likely to become long-term participants [42].

Most data collected in the LUCAS project were self-reported. There are advantages and disadvantages with this. Strategies to handle administrative staff training, data entry rules, minimising missing data, quality control, contacts and newsletters for follow-ups to maintain the relationship between participants and staff were previously described in [27,28,43]. Hard facts were collected as well: occurrence and time of death, entitlement for professional nursing care paid by the official German social insurance system and entry into nursing home.

Certain items were missing for some study participants due to dropout or incompleteness. The dropout rates up to the second and third wave were relatively low, and it appears that dropouts were more likely to die (Figure 3). This is supported by some anecdotal evidence from dropouts explaining their refusal due to exhaustion, cognitive problems, rapid health decline or entry into nursing home.

We did not collect medical data from the GPs or hospitals. In view of the German data protection laws, the collection of such data was judged to be a sensitive issue that might have jeopardised the project. Moreover, medical information was not in the focus of the LUCAS project that is concerned with health behaviour, function and normal ageing of initially healthy persons.

Unfortunately, we had no opportunity to collect genetics and epigenetic samples because of financial restrictions. In future, this would be indicated to get a better picture of still poorly understood interactions between environment, behaviour and biology [44].

## Conclusions

The LUCAS cohort provides epidemiological information about health during ageing. To accompany more than 3,300 cohort participants over a ten year period was an ambitious enterprise that required a tremendous coordinating effort including leadership, teamwork and excellent communication between the multidisciplinary project partners with expertise in the many health and contextual factors. In consequence, we achieved a relatively low rate of losses (not reached and refusals) of 28.9 % over ten years (about 3 % per year). Key components of this process were transparency and continuous integration of the senior citizens involved, the GPs, local and federal authorities as well as the project partners. The LUCAS cohort is expected to continue until 2013, at least.

We expect our data to help with the prediction of impending decline towards frailty as well as with identifying protective factors counteracting such decline. Description of symptoms indicating functional decline and its main causes may be an interesting product for GPs, clinicians, nurses and other professionals in geriatric care. The cohort design facilitated an RCT of a health intervention approach that is on its way into the regular health system in Germany. Currently, further embedded RCTs are under development within the LUCAS framework.

## Ethical approval

The ethical approval of the PRO-AGE project was given by the Ethics Committee of the Ärztekammer Hamburg (Germany) in 2000. The subsequent and ongoing LUCAS project phase was also approved by the Ethics Committee of the Ärztekammer Hamburg (Germany) in 2007.

## Competing interests

The author(s) declare that they have no competing interests.

## Authors' contributions

All authors are members of the PRO-AGE project group and the LUCAS consortium and participated in the conceptualization and implementation of the study. HMB and UD were the administrative coordinators of the PRO-AGE project. WRK is speaker of the LUCAS consortium, UD the administrative coordinator of the consortium and scientific coordinator of the longitudinal urban cohort. UD, JA, SG, WRK, HMB implemented the PRO-AGE and the ongoing LUCAS trial in Hamburg (Germany). SG was in charge of the LUCAS data management and tabulations. CM is senior consultant to the LUCAS project and contributed to the trial design, data analysis, data interpretation, and manuscript preparation. UD and CM developed the first and final drafts of this manuscript, and all authors contributed to it. All authors read and approved the final manuscript.

## Pre-publication history

The pre-publication history for this paper can be accessed here:

http://www.biomedcentral.com/1471-2318/12/35/prepub

## Supplementary Material

**Additional file 1: Table S1.** Domains covered in the self-administered questionnaires at baseline, wave 1, wave 2, and wave 3. Click here for file
