# Supplementary material for: The longitudinal urban cohort ageing study (LUCAS): study protocol and participation in the first decade
Source: BMC Geriatr. 2012 Jul 9;12:35. doi: 10.1186/1471-2318-12-35 (PMC3674861; doi:10.1186/1471-2318-12-35)
Supplement: Supplementary file 1 — Additional file 1: Table S1. Domains covered in the self-administered questionnaires at baseline, wave 1, wave 2, and wave 3. [file 1471-2318-12-35-S1.pdf]

Supplementary Table: Domains covered in the self-administered questionnaires at baseline, wave 1, wave 2, and wave 3

**Domains of interest (1-18)**	**Baseline Pra-questionnaire (2000-2001)**	**Wave 1 HRA-O (2000-2002)**	**Wave 2 LUCAS (2007-2008)**	**Wave 3 LUCAS (2009-2010)**
**1-About participant / socio-demographic factors**<br>e.g., age, sex, place of birth, income, native language, migration	2[*]	5[1]	4[1,2]	1[1]
**2-Alcohol and Tobacco Use**	-	18[1]	1[1]	1[1]
**3-Cognition**<br>e.g., cognitive activity	-	10[1]	0[1]	4[1]
**4-Environmental factors**<br>e.g., place of living, technical aids, access to garden or parks, access to public transport	1[*]	1[1]	1[1]	6[1]
**5-Functional Limitations**<br>e.g., B-ADL, I-ADL, urinary incontinence, independence in daily activities	2[3,4]	25[1]	12[1,5]	13[1,5]
**6-Health Care Use, Co- Morbidity and Medical History**<br>e.g., hospitalisation, physician visits, no. of diagnoses, ICF-short form	5	15[6,1]	4[6,1]	60[6,1,7]
**7-Health measurements and symptoms**<br>e.g., weight and height, BMI	-	39[1]	2[1]	2[1]
**8-Injury Prevention and Fall Syndrom**<br>e.g., fallrisk check, falls, consequences, fear of falling	-	7[1]	4[1,8]	4[1,8]
**9-Medication**<br>e.g., medical history, no. of medications, no. of OTC drugs	-	14[1]	3[1]	3[1]
**10-Mobility and Urban Activity Space**<br>e.g., motor vehicle use, public transport use, everyday life and leisure time	-	4[1]	3[1,2]	5[1,2]
**11-Nutrition and Oral Health**<br>e.g., nutrition record, weigth loss, oral health	-	39[1]	1[1]	1[1]

**Domains of interest (1-18)**	**Baseline Pra questionnaire (2000-2001)**	**Wave 1 HRA-O (2000-2002)**	**Wave 2 LUCAS (2007-2008)**	**Wave 3 LUCAS (2009-2010)**
**12-Pain**	-	26[1]	1[1]	3[1]
**13-Physical and Mental Activity**	-	22[1]	7[1,9]	8[1,9]
**14-Preventative and Health Improving measures** e.g., health checkup (GP), dental checkup, immunisations, attitude	-	11[1]	3[1]	5[1,3]
**15-Psychological factors and Affect** e.g., depressive mood, attitude of ageing, health literacy	-	23[1]	1[1]	9[1,3]
**16-Self-Perceived Health and Quality of Life**	1	1[6,1]	1[1]	2[1,3]
**17-Sensory Systems** e.g., vision problems, hearing problems	-	22[1]	4[1]	4[1]
**18-Social factors and Life events** e.g., social net, social activity, education, profession, biography	1	17[6,1]	8[6,1,3]	11[6,3,1]

*** Age, sex and address are covered in all waves but shown in this table 1 only once at baseline**

Domains are related to functional decline in community-dwelling elderly people according to a systematic review: Stuck AE, Walthert JM, Nikolaus T, Büla CJ, Hohmann C, Beck JC: **Risk factors for functional status decline in community-living elderly people: A systematic literature review.** *Soc Sci Med* 1999, 48:445-469 and a frailty framework: Bergman H, Beland F, Karunananthan S, Hummel S, Hogan D, Wolfson C: **Developing a Working Framework for Understanding Frailty 2004**. [http://www.frail-fragile.ca].

References to individual questions:

[1]Stuck AE, Kharicha K, Dapp U, Anders J, von Renteln-Kruse W, Meier-Baumgartner HP, Harari D, Swift CG, Ivanova K, Egger M, Gillmann G, Higa J, Beck JC,. Iliffe S: **Development, feasibility and performance of a health risk appraisal questionnaire for older persons.** *BMC Medical Research Methods* 2007, **7:**1 (online, open access).

[2]Dapp U, Lorentz Ch, Laub S, Anders J, von Renteln-Kruse W, Minder Ch, Dirksen-Fischer M: **Im Alter aktiv und gesund leben – Ergebnisse einer repräsentativen Seniorenbefragung in Hamburg.** *Z Gerontol Geriat* 2009; **42:**245-255.

[3]Own development, e.g. question to “Pfegestufe” according to the German health insurance law (SGB XI Soziale Pflegeversicherung. 10. Auflage, Taschenbuch Verlag 2010).

[4]Katz S, Ford AB, Moskowitz RW, Jackson BA, Jaffe MW: **Studies of illness in the aged. The index of ADL: A standardized measure of biological and psychosocial function.** *JAMA* 1963, 185:914-919.

[5]DEGAM – Deutsche Gesellschaft für Allgemeinmedizin und Familienmedizin*: Leitlinie 5: Harninkontinenz.* Düsseldorf; 2004. [http//:www.degam-leitlinien.de]

[6]Boult C, Dowd B, McCaffrey D, Boult L, Hernandez R, Krulewitch H: **Screening elders for risk of hospital admission.** *J Am Geriatr Soc* 1993, **41:**811-817.

[7]WHO – World Health Organization: *ICF Checklist, Version 2.1a, Clinician Form for International Classification of Functioning, Disability and Health.* 2003. [http://www.who.int/classifications/icf/en/]

[8]Anders J, Dapp U, Laub S, von Renteln-Kruse W, Juhl K: **Einschätzung der Sturzgefährdung gebrechlicher, noch selbstständig lebender, älterer Menschen.** *Z Gerontol Geriat* 2006, **39:**268-276.

[9]Meier-Baumgartner HP, Dapp U, Anders J: *Aktive Gesundheitsförderung im Alter: Ein neuartiges Präventionsprogramm für Senioren.* 2nd. edition. Stuttgart: Kohlhammer Verlag; 2006.
